# Initial Dose of Three Monthly Intravitreal Injections versus PRN Intravitreal Injections of Bevacizumab for Macular Edema Secondary to Branch Retinal Vein Occlusion

**DOI:** 10.1155/2013/209735

**Published:** 2013-08-26

**Authors:** Seong Joon Ahn, Jeeyun Ahn, Se Joon Woo, Kyu Hyung Park

**Affiliations:** ^1^Department of Ophthalmology, College of Medicine, Seoul National University Bundang Hospital, 166 Gumiro, Bundang-gu, Seongnam, Gyeonggi-do 463-707, Republic of Korea; ^2^Department of Ophthalmology, Seoul National University-Seoul Metropolitan Government Boramae Medical Center, 20 Boramae-ro 5 gil, Dongjak-gu, Seoul 156-707, Republic of Korea

## Abstract

*Purpose*. To compare visual and anatomic outcomes of intravitreal bevacizumab injections administered as needed (PRN group) and initial treatment with 3 monthly injections followed by as-needed injections (3 monthly initial dose group) in patients with macular edema (ME) secondary to branch retinal vein occlusion (BRVO). *Methods*. This retrospective study included 69 and 26 patients in the PRN and 3 monthly initial dose groups, respectively. Best-corrected visual acuity (BCVA) and central retinal thickness (CRT) were compared between the 2 groups 6 months after initial injection. *Results*. At month 6, BCVA change from baseline was −0.27 ± 0.28 (mean ± standard deviation) logMAR in the PRN group and −0.28 ± 0.20 logMAR in the 3 monthly initial dose group. Mean CRT changes were −204 ± 168 in the PRN group and −161 ± 149 **μ**m in the 3 monthly initial dose group at month 6. There were no statistically significant differences in BCVA or CRT changes between groups at any time point. The number of intravitreal injections over 6 months was significantly lower in the PRN group (1.8 ± 0.8 injections) than in the 3 monthly initial dose group (3.4 ± 0.5 injections; *P* < 0.001). *Conclusions*. Our results suggest that as-needed intravitreal bevacizumab injections are more tolerable for patients with ME secondary to BRVO.

## 1. Introduction

Macular edema (ME) is one of the most common causes of visual loss in patients with retinal vein occlusions (RVOs) [1]. Several studies have reported the efficacy of diverse treatment modalities for this condition. For example, the randomized controlled branch vein occlusion study (BVOS) revealed that patients treated with grid photocoagulation showed a greater visual improvement than did untreated patients [2]. The standard care versus corticosteroid for retinal vein occlusion (SCORE) study revealed that at 12 months, there was no difference in visual acuity between patients treated with standard care (grid photocoagulation) and those treated with intravitreal injection of the corticosteroid triamcinolone acetonide [3]. Additionally, surgical treatment (vitrectomy with internal limiting membrane peeling) results in visual improvement in patients with ME secondary to either central RVO (CRVO) or branch RVO (BRVO) [4, 5].

Vascular endothelial growth factor (VEGF) inhibitors also facilitate the resolution of ME resulting from RVO. Vascular occlusion induces VEGF upregulation, leading to increased vascular permeability and subsequent ME [6–8]. Additionally, the degree of ME is correlated with VEGF levels in the aqueous humour of eyes with RVO [6]. Therefore, it is not surprising that recent clinical studies have reported successful results with intravitreal VEGF antagonists for the treatment of ME resulting from RVO [9–11].

Among clinically used VEGF antagonists, ranibizumab (Lucentis, Genentech, Inc., San Francisco, CA, USA) was the first to be approved by the Food and Drug Administration (FDA) for the treatment of ME following RVO. This approval was based on data from the Branch retinal vein occlusion (BRAVO): Evaluation of efficacy and safety study, which showed that intraocular injections of ranibizumab provide an effective treatment for ME after BRVO [10, 11]. However, intravitreal anti-VEGF agents only induce transient ME improvement [12, 13], and repeated injections are usually necessary [14–20], In the BRAVO study, patients with BRVO received monthly intraocular ranibizumab injections for 6 months [10, 11]. Due to cost constraints (>$1000 per treatment), off-label use of intravitreal bevacizumab (Avastin, Genentech, San Francisco, CA, USA) is widely used to treat patients with ME secondary to RVO. Previous studies evaluating various dosing regimens have shown the efficacy of bevacizumab for treating ME due to RVO [15, 19, 21–23]; however, an optimal bevacizumab injection dosing schedule has yet to be determined.

This study examined 2 different intravitreal bevacizumab injection dosing schedules in patients with ME secondary to BRVO. Previous reports have shown successful outcomes with bevacizumab treatment in patients with RVO following either an as-needed dosing schedule [15] or 3 monthly injections followed by as-needed dosing [19]. Herein, we compare these 2 treatment schedules in a single-center retrospective study.

## 2. Materials and Methods

### 2.1. Patient Selection and Treatment Groups

This retrospective study included patients diagnosed with ME secondary to BRVO at Seoul National University Bundang Hospital from July 2009 to December 2011. This study was approved by the institutional review board and adhered to the tenets of the Declaration of Helsinki. Informed consent was obtained from all patients after a thorough discussion about the potential benefits and risks of bevacizumab injections.

Inclusion criteria were as follows: (1) best-corrected visual acuity (BCVA) of less than 20/30, (2) symptom duration less than 6 months, (3) central retinal thickness (CRT) > 300 *μ*m, as measured by optical coherence tomography (OCT), and (4) patient follow-up for ≥6 months after the first intravitreal bevacizumab injection. Patients with other ocular conditions associated with ME or increased intraocular VEGF levels (e.g., uveitis, neovascular glaucoma, exudative age-related macular degeneration, diabetic macular edema, or ocular ischemic syndrome) were excluded from analyses. Furthermore, patients who had a history of previous treatment with intravitreal triamcinolone acetonide or anti-VEGF agents were excluded. Patients who underwent cataract extraction or other ocular procedures during the follow-up period were also excluded.

Intravitreal bevacizumab injections were administered by 2 retina specialists (KHP and SJW) using the same procedures: topical anaesthetic drops were administered, the injection site was washed with 5% povidone iodine, a lid speculum was inserted, a 30-gauge needle was inserted through the pars plana, and bevacizumab (Avastin, 1.25 mg in 0.05 mL) was injected into the vitreous. Patients were divided into 2 subgroups according to dosing regimen. In the PRN group, patients were treated on an as-needed basis after a single injection. Retreatment was administered if (1) the patient had BCVA worse than 20/40 or (2) CRT was more than 300 *μ*m. In the 3 monthly initial dose group, patients received 3 initial intravitreal bevacizumab injections at monthly intervals, regardless of the visual and anatomic outcomes at months 1 and 2. After the initial injections, retreatment was performed using the same criteria as the PRN group. Three monthly injections as initial dose were performed for the patients with ME secondary to BRVO before June 2010 but afterwards, the dosing regimen was altered to PRN injections. 

Laser treatment was not combined with the initial injection of bevacizumab for any patient. Starting at month 3, rescue laser photocoagulation was performed for patients with refractory ME in whom repeated bevacizumab injections failed to improve BCVA or decrease CRT if hemorrhages in the macula had cleared sufficiently to perform grid laser photocoagulation. This was necessary in 13 (18.8%) eyes from the PRN group and in 6 eyes (20.7%) from the 3 monthly initial dose group. This difference was not statistically significant (*P* = 0.65, chi-square test).

### 2.2. Evaluation of Baseline Status and Treatment Outcome

All patients underwent a complete eye examination, including BCVA, slit-lamp biomicroscopy, intraocular pressure, and dilated fundus examination at the visit before the initial injection. ME was assessed using Spectralis OCT (Heidelberg Engineering, Heidelberg, Germany). CRT was measured using a circular map analysis protocol, which measures the distance between the first signal from the vitreoretinal interface and the signal from the outer border of the retinal pigment epithelium and calculates the average thickness in a 1 mm diameter circle centerd on the fovea. At baseline, photoreceptor status was evaluated using OCT by investigating morphologic abnormalities in the inner segment-outer segment (IS-OS) line, which shows an association with visual outcome [24, 25]. The presence of serous macular detachment, which may also be a predictive factor for ranibizumab treatment outcome [26], was assessed using baseline OCT images of the fovea. 

 Fluorescein angiography (FA) and fundus photography were also performed at baseline. The percentage area of retinal hemorrhage in a circle with a radius of 3600 *μ*m centerd on the fovea was calculated. NIH ImageJ software (ImageJ 1.44p, National Institutes of Health, Bethesda, MD, USA) was used to draw a polygon within the circle along the margins of the retinal hemorrhage. The area of the polygon was measured and then divided by the area of the circle to determine the percentage of the area of retinal hemorrhage. The same software was used to calculate the percentage of the area of retinal ischemia. Capillary nonperfusion, defined as dropout of the retinal capillary bed, was detected on FA. False dropout resulting from blockage of fluorescence by the hemorrhage was distinguished from capillary nonperfusion by comparing the FA image with fundus photographs. The nonperfused area was divided by the optic disc area to calculate the severity of retinal ischemia. Ischemic BRVO was defined as the nonperfused area divided by the disc area ≥5 [27]. Foveal nonperfusion was defined as the extent of capillary dropout on the fovea in >1 quadrant.

Patients were examined monthly for 6 months and then at least every 3 months, and qualified technicians masked to patients' information measured BCVA in the same room using a Snellen chart. OCT and fundus photography were performed at each visit, and FA was performed at months 3 and 6. BCVA and CRT were reviewed for 6 months, and the change from baseline was calculated at each follow-up visit. If available, the data on BCVA and CRT at 1 year after the initial injection of bevacizumab were also retrieved from medical charts and used for analyses.

For visual outcome comparisons, the percentage of patients who gained 2 or more Snellen lines at month 6 was calculated for each group. In both groups, the percentages of patients who improved 1, 2, or ≥3 Snellen lines were assessed at months 1, 3, and 6. For anatomic outcome, the percentage of patients who had complete ME resolution was obtained in each group and compared between groups. Complete resolution was defined as a CRT of <270 *μ*m without morphologic abnormalities associated with ME on OCT images (e.g., intraretinal cyst and subretinal fluid). Additionally, the incidence of ME recurrence (CRT increases to >300 *μ*m) during the 6-month period was compared between groups.

The absorption of retinal hemorrhage and changes in nonperfusion area from baseline were compared between the 2 groups at months 3 and 6. For the absorption of retinal hemorrhage, the changes at month 1 were also compared between groups. 

In addition, as little is known about the influence of retinal ischemia on the response to anti-VEGF therapy in patients with BRVO, we also evaluated the influence of retinal ischemia on visual gain and anatomical improvement by comparing BCVA change and CRT change between eyes with ischemic and those with nonischemic BRVO. The number of anti-VEGF injections was also compared between the two groups.

### 2.3. Statistical Analysis

The mean changes in BCVA and CRT from baseline were compared between groups using Student's *t*-tests at each month. The data on retinal hemorrhage and nonperfusion area were compared using the same test. Frequency and incidence data were compared using chi-square or Fisher's exact test. Data for continuous variables is expressed as mean values ± standard deviation. *P* values <0.05 were considered statistically significant, and those between 0.05 and 0.10 were considered marginally significant. Statistical analyses were performed using SPSS for Windows (Ver. 17.0, Statistical Package for the Social Sciences, SPSS Inc., Chicago, IL, USA).

## 3. Results

### 3.1. Patient Characteristics and Number of Injections

We reviewed the medical charts of 95 eyes from 94 patients (44 male and 50 female) with macular edema secondary to BRVO. All patients had follow-up periods of ≥6 months, and 47 patients in the PRN group and 16 patients in the 3 monthly initial dose group had follow-up periods of ≥1 year. Patient characteristics and clinical features of the 2 groups are summarized in Table 1. Patient demographics and baseline ocular characteristics were similar across treatment groups. In particular, the proportion of ischemic BRVO was 16 of 26 (61.5%) in the 3 monthly initial dose group and 38 of 69 (55.1%) in the PRN group, which was not significantly different (*P* = 0.572, chi-square test).

At month 6, patients in the PRN group had received significantly fewer injections than those in the 3 monthly initial dose group (1.8 ± 0.8 (range, 1–4) versus 3.4 ± 0.5 (range, 3-4) injections on average; *P* < 0.001, Student's *t*-test). The numbers of injections over 6 months were 2.1 ± 1.1 in the eyes with nonischemic BRVO and 2.4 ± 1.0 in those with ischemic BRVO, respectively, showing insignificant difference between the two groups (*P* = 0.164, Student's *t* test). The mean numbers of injections over the 1-year period were 2.3 ± 1.3 (range, 1–6) in the PRN group and 3.8 ± 1.0 (range, 3–7) in the 3 monthly initial dose group, respectively, (*P* < 0.001, Student's *t*-test). No serious complications, such as endophthalmitis, vitreous hemorrhage, uveitis, or retinal detachment, developed in either group.

### 3.2. Visual Outcomes

**Figure 1:**
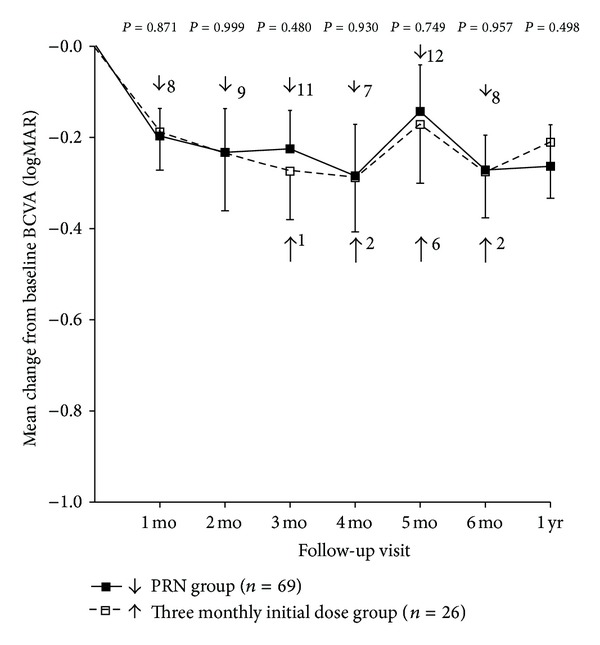
Mean best-corrected visual acuity (BCVA) change from baseline to 12 months. There was no significant difference in BCVA changes between the PRN group and the 3 monthly initial dose group at all postoperative visits. Error bars denote upper or lower bound of 95% confidence intervals. Statistical analyses were performed using Student's *t*-tests. The numbers on the right side of the arrow indicate the number of patients who were retreated with intravitreal bevacizumab injections at that time.

**Figure 2:**
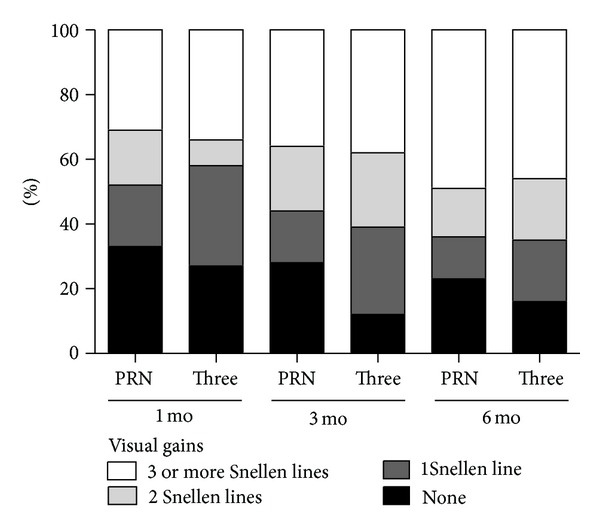
Visual gains and their percentages at months 1, 3, and 6. The incidences of eyes showing visual gain (1, 2, or ≥3 Snellen lines) are generally similar between the 2 dosing regimens; however, at month 3, the percentages of eyes that gained 1, 2, and ≥3 Snellen lines were greater in the 3 monthly initial dose group than in the PRN group.

Bevacizumab treatment resulted in significant BCVA improvement in both groups. Mean BCVA of the PRN group was 0.61 ± 0.35 logMAR at baseline and 0.35 ± 0.30 logMAR (*P* < 0.001, paired *t* test) at month 6. Mean BCVA of the 3 monthly initial dose group was 0.65 ± 0.34 logMAR at baseline and 0.36 ± 0.29 logMAR at month 6 (*P* < 0.001, paired *t* test). Mean BCVA showed marked improvement at month 1 and only moderate improvement at month 2 in both groups. However, at month 3, the PRN group showed worsening of mean BCVA compared to month 2. This was not the case in the 3 monthly initial dose group, which showed additional improvement in BCVA after the third injection at month 2. Worsening of mean BCVA was also noted in the 3 monthly initial dose group at month 5 (Figure 1). The percentage of patients who showed improvement of 1, 2, or ≥3 Snellen lines after bevacizumab treatment is presented in Figure 2. The incidence of eyes that gained 1, 2, and ≥3 Snellen lines was generally similar between the 2 groups except at month 3, when the percentages of eyes showing visual gains were greater in the 3 monthly initial dose group than in the PRN group.

Between groups, there was no significant difference in mean BCVA change from baseline to any time point examined. At month 6, 44 of 69 eyes (63.8%) in the PRN group and 17 of 26 eyes (65.4%) in the 3 monthly initial dose group had visual improvement of 2 Snellen lines or greater. The difference between groups was not statistically significant (*P* = 0.89, chi-square test; Table 2). Mean BCVA changes were −0.25 ± 0.24 and −0.29 ± 0.28 logMAR in the eyes with nonischemic and ischemic BRVO, respectively, (*P* = 0.577, Student's *t*-test).

### 3.3. Anatomic Outcomes

**Figure 3:**
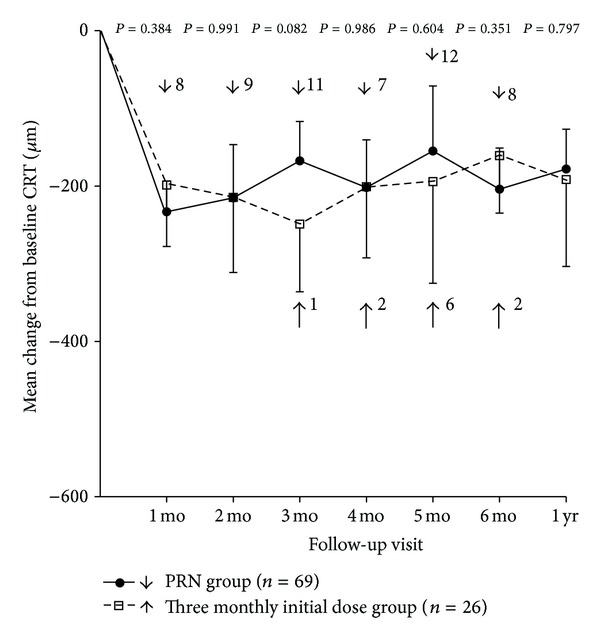
Mean central retinal thickness (CRT) change from baseline over time to 12 months after the initial bevacizumab injection. There is no significant difference in CRT change between the 2 treatment schedules at any visit. Only at month 3, CRT change showed a marginally significant difference between the 2 groups. Error bars denote upper or lower bound of 95% confidence intervals. Statistical analyses were performed using Student's *t*-tests. The numbers on the right side of the arrow indicate the numbers of patients who were retreated with intravitreal bevacizumab injection at that time.

Each group showed a reduction in CRT from baseline at all monthly visits (Figure 3). At month 6, the mean changes in CRT from baseline were −204 ± 168 *μ*m in the PRN group and −161 ± 149 *μ*m in the 3 monthly initial dose group. At all follow-up visits, there was no significant difference in mean CRT change between the 2 groups. Only a marginally significant difference was observed at month 3, when CRT decreased more from baseline in the 3 monthly initial dose group than in the PRN group (−249 ± 169 versus −167 ± 149 *μ*m; *P* = 0.082, Student's *t*-test). Mean CRT changes were −159 ± 170 and −211 ± 156 *μ*m in the non-ischemic and ischemic groups, respectively, which was not significantly different (*P* = 0.231, Student's *t* test).

 The frequency of complete ME resolution at month 6 was not significantly different between groups (58.0% (40 of 69) eyes in the PRN group versus 50.0% [13 of 26] eyes in the 3 monthly initial dose group; *P* = 0.50, chi-square test). The frequency of cases with recurrence over the 6-month period was 60.9% (42 of 69 eyes) in the PRN group and 46.2% (12 of 26 eyes) in the 3 monthly initial dose group (Table 2); this difference was not statistically significant (*P* = 0.20, chi-square test).

The absorption of retinal hemorrhage and the changes in nonperfusion areas were compared between the 2 groups. The area of hemorrhage absorption was significantly different between groups at month 3 (9.3% ± 10.4% (PRN group) versus 16.2% ± 10.5% (3 monthly initial dose group) of the area of the 3600 *μ*m radius circle; *P* = 0.027, Student's *t*-test). However, there was no significant difference at month 1 (6.2% ± 5.3% (PRN group) versus 6.6% ± 6.0% (3 monthly initial dose group) of the area of the 3600 *μ*m radius circle; *P* = 0.80, Student's *t*-test) and month 6 (15.1% ± 13.9% (PRN group) versus 18.3% ± 13.1% (3 monthly initial dose group); *P* = 0.41). The change in nonperfusion area at month 3 was −0.1% ± 2.0% in the PRN group and −0.8% ± 1.8% in the 3 monthly initial dose group. The nonperfusion areas at month 3 were not significantly different from baseline for either group (*P* = 0.71 and 0.27 in the PRN and 3 monthly initial dose groups, resp. paired *t*-test). Furthermore, the changes were not significantly different between the 2 groups (*P* = 0.46, Student's *t*-test). The change at month 6 was 0.4% ± 3.3% in the PRN group and −0.3% ± 3.2% in the 3 monthly initial dose group. These changes were not significantly different from baseline in either group (*P* = 0.49 (PRN group) and 0.74 (3 monthly initial dose group)) or from each other (*P* = 0.50, Student's *t*-test).

## 4. Discussion

The optimal bevacizumab treatment dosing schedule for ME secondary to BRVO has not yet been determined. As 6 monthly initial injections used in the BRAVO study [9, 28] are challenging for patients, as-needed injections or 3 monthly initial doses followed by as-needed injections may be more practical alternatives. This study demonstrated that there was no statistical difference in visual or anatomic outcomes between as-needed treatment and 3 monthly initial doses followed by as-needed injections for 6 months and at 1 year after the therapy. This study suggests that the efficacy of the 2 dosing schedules may be equivalent for the time periods evaluated. More specifically, visual acuity changes were not significantly different between the 2 groups for 6 months after initiating bevacizumab treatment, and similar percentages of patients in both groups gained 2 or more Snellen lines at month 6. Anatomic outcomes, such as CRT change, complete ME resolution, and recurrence over the 6-month period, were not significantly different between groups. However, the mean number of injections administered over the study period was significantly lower in the PRN group than in the 3 monthly initial dose group. This suggests that as-needed bevacizumab injections are more tolerable for patients with ME secondary to BRVO.

In previous studies, BCVA improvement varied from 0.26 to 0.44 logMAR units 6 months after treatment with bevacizumab in patients with ME secondary to RVO [15–17]. The results are comparable with ours, which showed an improvement of 0.27 logMAR units in the PRN group and 0.28 logMAR units in the 3 monthly initial dose group at month 6. CRT decreased by 204 *μ*m in the PRN group and 161 *μ*m in the 3 monthly initial dose group; these findings were also similar to the 184–218.7 *μ*m reduction observed in previous studies [15, 16, 19]. Although the mean number of injections during 1-year period varied in different studies, namely, 2.3 and 3.8, BCVA and CRT showed comparable results in all studies.

 As an anatomic measure, areas of retinal hemorrhage and ischemia were compared between the 2 dosing schedules. Hemorrhage covering the macula can affect treatment decisions, as the hemorrhage should be sufficiently cleared for safe laser application. Faster absorption of hemorrhages might improve visual outcome by allowing laser treatment to be performed earlier in patients with recurrent or refractory ME. In the BRAVO study, the number of patients without intraretinal hemorrhage was higher in the treatment group than in the sham group after 6 months of treatment [11]. In our study, the 3 monthly initial dose group showed significantly greater absorption at month 3 than did the PRN group. These results indicate that bevacizumab treatment facilitates the absorption of retinal hemorrhage in eyes with BRVO, depending on the number of injections. Despite the potential benefit of faster absorption of retinal hemorrhage, visual outcome was not significantly better in the 3 monthly initial dose group.

A controversial issue regarding intravitreal bevacizumab use centers on whether it increases retinal ischemia [29]. Terui et al. showed that changes in the capillary nonperfusion area were insignificant 1 month after intravitreal bevacizumab injection [29]. This result is compatible with ours, as the areas of capillary nonperfusion were not significantly changed after injection in our study. Our data also showed that the changes at 3 and 6 months after the injection were not significantly different between the 2 treatment schedules. Collectively, these data suggest that intravitreal bevacizumab injections do not aggravate retinal ischemia in patients with BRVO. 

We also showed that ME regression mainly occurred after the initial injection in both groups. Further injections, in contrast, had much smaller effects on CRT. A similar pattern of changes in macular thickness after intravitreal bevacizumab treatment has also been noted in previous studies [15–19]. On the basis of this finding, we consider that the second or third monthly injection mainly maintains reduced CRT, rather than inducing significant CRT reduction. We therefore suggest that additional treatment is not required 1 month after the initial injection if the patients have attained complete resolution at that time. A recent report from the HORIZON trial showed that reduced follow-up and fewer ranibizumab injections in the second year of treatment were not observed in conjunction with significant worsening of mean BCVA and CRT among patients with ME secondary to BRVO [30]. In the aforementioned study, the visual acuity gains in patients with BRVO were maintained after 12 months of treatment, despite the reduced frequency of injections [30]. When initial intravitreal bevacizumab injection produces complete ME resolution, we believe that an as-needed treatment, rather than additional loading dose injections, may be sufficient and tolerable for patients with BRVO. In general, our study suggests that more tailored and personalized dosing regimens are more appropriate for treating ME secondary to BRVO.

Some limitations of our study should be considered. First, this is a retrospective study that has intrinsic drawbacks regarding bias. Second, a small number of patients were included in this study, and only 2 intravitreal bevacizumab injection dosing schedules were compared. Further comparative studies are required to determine the most appropriate dosing schedule of intravitreal bevacizumab injections for BRVO patients with ME. Another limitation is that our study adopted Snellen chart for BCVA assessment, which has well-documented limitations such as inconsistent progression in letter size from one line to another and unequal legibility of letters used [31].

In summary, there were no significant differences in visual or anatomic outcomes between patients treated with as-needed bevacizumab treatment and those treated with 3 monthly initial doses followed by PRN injections. Treatment on an as-needed basis reduced the number of injections without significantly compromising efficacy. The results of this study may be helpful for clinicians to determine an intravitreal bevacizumab injection dosing regimen for ME secondary to BRVO. Randomized studies with longer follow-up periods are required to confirm our findings.

## Figures and Tables

**Table 1:** Patient demographics and baseline ocular characteristics in the PRN and 3 monthly initial dose groups.

	PRN group (*n* = 69)	Three monthly initial dose group (*n* = 26)	*P*
Age	61.9 ± 12.3	60.0 ± 8.4	0.46
Sex: male, female	32 : 37	13 : 13	0.75
Diabetes mellitus (%)	4 (5.8%)	4 (15.4%)	0.21
Hypertension (%)	30 (43.5%)	11 (42.3%)	0.92
Location of retinal vein occlusion, ST BRVO : IT BRVO	40 : 29	18 : 8	0.32
Pretreatment visual acuity, log MAR	0.61 ± 0.35 (range: 0.22–1.7)	0.65 ± 0.34 (range: 0.22–1.4)	0.61
Ischemic* BRVO: non-ischemic BRVO	38 : 31	16 : 10	0.57
OCT findings			
Pretreatment central retinal thickness, *μ*m	510 ± 150	528 ± 124	0.58
Photoreceptor IS-OS status, Normal : disrupted (%)	21 : 48 (30.4% : 69.6%)	8 : 18 (30.8% : 69.2%)	1.0
Photoreceptor ELM status, Normal : disrupted (%)	37 : 32 (53.6% : 46.4%)	14 : 12 (53.8% : 46.2%)	1.0
Serous macular detachment (%)	34 (49.3%)	14 (53.8%)	0.69
Funduscopic and angiographic findings			
Foveal nonperfusion (%)^†^	16 (23.2%)	5 (19.2%)	0.68
Area of retinal hemorrhage, %^‡^	16.0 ± 12.7	21.1 ± 14.1	0.10
Area of capillary nonperfusion, %^‡^	29.8 ± 13.6	28.7 ± 17.1	0.76

BRVO: branch retinal vein occlusion; ELM: external limiting membrane; IS-OS: inner segment-outer segment; IT: inferior temporal; ST: superior temporal.

*P* value was obtained by student's *t*-test for continuous variables and chi-square or Fisher's exact test for dichotomous variables.

*Nonperfused area divided by the optic disc area ≥5.

^†^Foveal nonperfusion was defined as the extent of capillary dropout on the fovea for >1 quadrant.

^‡^The area of retinal hemorrhage or capillary nonperfusion was measured in a circle with a radius of 3600 *μ*m centred on the fovea using Image*J* software. By dividing this area by the area of the circle with a radius of 3600 *μ*m, the percentage of the area of retinal hemorrhage or capillary nonperfusion was calculated.

**Table 2:** Visual and anatomic outcomes following bevacizumab injection.

	PRN group (*n* = 69)	Three monthly initial dose group (*n* = 26)	P*
Visual improvement at month 6 (%)	44 (63.8%)	17 (65.4%)	0.89
Complete resolution at month 6 (%)	40 (58.0%)	13 (50.0%)	0.50
Recurrence over 6-month period (%)	42 (60.9%)	12 (46.2%)	0.20
Absorption of retinal hemorrhage^†^			
Month 3, %	9.3 ± 10.4	16.2 ± 10.5	**0.027**
Month 6, %	15.1 ± 13.9	18.3 ± 13.1	0.41
Change of nonperfusion area^†^			
Month 3, %	−0.1 ± 2.0^‡^	−0.8 ± 1.8^‡^	0.46
Month 6, %	0.4 ± 3.3^‡^	−0.3 ± 3.26^‡^	0.50

*P values were obtained by Student's *t*-test for continuous variables and chi-square test for dichotomous variables.

^†^Percentages were calculated by dividing the area of hemorrhage absorption or change in nonperfusion areas by the area of the circle with a radius of 3600 *μ*m.

^‡^Negative numbers indicate the decrease in the area of nonperfusion during the time period.

Boldface indicates statistical significance (*P* < 0.05).
